# Belgium experience in disaster victim identification applied in handling terrorist attack at Brussels Airport 2016

**DOI:** 10.1080/20961790.2020.1775932

**Published:** 2020-11-02

**Authors:** François Beauthier, Wim Van de Voorde, Philippe Lefevre, Jean-Pol Beauthier

**Affiliations:** a Medicolegal and Forensic Anthropology Unit, Laboratory of Anatomy, Biomechanics and Organogenesis, Faculty of Medicine, Université libre de Bruxelles, Bruxelles, Belgium; b Forensic Biomedical Sciences, Department of Imaging and Pathology, University of Leuven, Belgium

**Keywords:** Forensic sciences, forensic pathology, terrorist attack, blast injury, body fragments, identification

## Abstract

The Belgian disaster victim identification (DVI) team is involved in many investigations in our country. Indeed, this specialized team of the federal police oversees searching for and investigating criminally buried dead bodies, identification of unknown putrefied corpses, and more. The Belgian DVI team also assists with the identification of victims of mass disasters (natural, accidental, and mass murders). In this article, we consider the contributions of different teams (forensic pathology, anthropology, and odontology, federal police, and crime scene investigation) both on the scene of the attack at the Brussels National Airport (Zaventem) and in the laboratory work (autopsies, sample studies).

## Introduction

Interventions conducted by the Disaster Victim Identification (DVI) Belgium are quite varied in our country. This specialized team of federal police is involved in searching for buried corpses, the investigations of unidentified degraded bodies, and cases of criminal excavation. This service is also involved in the identification process for victims of disasters (natural, accidental, and mass murders). The identification of an unknown person is a complex process involving individuals with various skills in the field (crime scene investigators, forensic pathologists, anthropologists, and odontologists). The Belgian DVI team coordinates the management of serious accidents (e.g. house collapses, aircraft and train accidents), natural disasters (e.g. the 2004 tsunami in Thailand [1–3]), and mass civilian killings (e.g. during the conflict in Kosovo several years ago) [4]. In addition to the specific missions for isolated identification of individuals, particularly in the context of criminal disappearances [5], Belgian teams have partaken in the identification process of victims involved in cases of terrorist attacks (e.g. the MH17 crash after missile hit in Ukraine, and bombing attacks in Brussels and Zaventem in Belgium).

## Circumstances

The attacks in Brussels on 22 March 2016 took place 7 days after Forest's police operations on 15 March 2016, which led to the arrest of Salah Abdeslam 3 days later in Molenbeek (one of the 19 towns of Brussels). This arrest followed the investigative procedure of the attacks that occurred on 13 November 2015 in France.

On 22 March 2016 at 7:58 am, two explosions occurred in the departures hall of Brussels International Airport in Zaventem, Flemish Brabant: one near Brussels Airlines reception and the other near the desk of American Airlines, where many passengers had checked their luggage for a flight to New York. The disaster plan was initiated as a result of this double explosion. The coordinating committee for threat analysis then decided to suspend all air traffic at the airport and raise the alert level to level 4 (highest level) for the entire country. Another explosion occurred at 9:11 am in the Brussels metro on a train leaving the Maelbeek station (in the European district) towards the City Centre. Following this disaster, 32 people were dead (not including the three suicide bombers) and more than 340 were wounded. Some of these wounded individuals are still undergoing heavy physical rehabilitation treatments in Belgium and neighbouring countries. In this article, we will discuss the management of bodies of people who were killed in the Zaventem Airport bombing.

### Engagement of the Department Forensic Medicine, University Hospitals Leuven

The head of the department, on his way to a homicide investigation, was alarmed by the first announcement of the explosions at the airport on the radio at 8:15 am on Tuesday, 22 March 2016 (Day 0). At 8:16 am, the prosecutor responsible for the Brussels Airport instructed our forensic pathologist on duty to rejoin the DVI and the scientific police at the airport for crime scene investigation. At 8:20 am, we informed the coordinator of our University Hospital about the situation and activated the disaster plan designed for a catastrophe involving dozens of dead victims. The Coordinator of Forensic Medicine (the head of the department) appointed a “Recovery of Corpses” Coordinator to engage in crime scene investigation, as well as a “Forensic Identification and Mortuary” Coordinator to manage the identification process in the morgue in cooperation with the DVI postmortem (PM) Coordinator. Their duties were described in corresponding action charts according to standard the International Criminal Police Organization (INTERPOL) procedures (https://www.interpol.int/How-we-work/Forensics/Disaster-Victim-Identification-DVI). Preparations were started to organize and prepare both the forensic medicine department and morgue to take in the bodies, examine them, use the three available autopsy rooms, and utilize the full body postmortem computed tomographies (PMCTs) in the radiology department.

The head of the department, as a member of the hospital incident coordination cell, was also involved in the development of the global disaster plan for the hospital, which was also involved in the care of surviving victims. The Forensic Identification and Mortuary Coordinator was continuously in contact with the Medical and Non-Medical Incident Manager of the hospital.

## Organization and methods

The methods are described for our various missions, which are adaptable depending on the circumstances, such as a buried *versus* unburied body [1, 6].

### The teams

The Belgian DVI is simply characterized by two teams with independent and separate activities:

- The antemortem (AM) team is in charge with the relatives for collecting all the useful elements in terms of identification such as physical parameters of the victims — weight, height — description of the clothes worn, scars, anatomical anomalies, deformities, old fractures, tattoos, collection of possible DNA samples from comb, toothbrush, etc. This team may be supported by members of the Red Cross or by psychologists or social workers [1];
- The PM team handles the recovery of the bodies, their examinations (external examinations and autopsies), the collection of all elements leading to physical identification (see above), biological samples (toxicology, DNA), and useful trace evidence for understanding the lethal mechanisms.

### Necrosearch and recovery — generalities

The essential grid techniques were adopted, as well as the numbering used in all operations of the same kind with e.g. crime scenes and non-identified dead bodies or scattered skeletal remains [1, 7, 8]:

- Zone numbering (Z_1_ to Z_n_), usually 10 m^2^;
- Numbering of neighbourhoods (Q_1_ to Q_n_);
- MPO labelling: body (M_1_ to M_n_), body parts (P_1_ to P_n_), and objects (O_1_ to O_n_), including all suspected elements related to explosive devices (in this case, homemade bombs with nails, screws bolts, and other metal objects).

In this particular case in Zaventem, the fragments were transported in bags numbered according to the aforementioned DVI technique, placed in containers also called and labelled (BAK_A_ to BAK_J_).

The numbering of the forensic laboratory has taken over the same numbering of the pieces (P_1_ to P_n_).

The traceability of the samples was carried out with the greatest care and with strict respect to classic DVI work, established from our various previous missions [8].

Strict compliance with recovery techniques is fundamental to the pursuit of quality identification. We previously described the enormous difficulties encountered when the recovery of bodies and fragments was carried out by unprofessional volunteers without respect to the procedure [1, 2].

### Details of the operation

#### Crime scene investigation

On Day 0, two forensic pathologists and one medical doctor studying forensic pathology arrived at the crime scene at 9:10 am. They began the search and examination of the corpses together with members of the DVI and crime scene investigators from 9:15 am until midnight. Additionally, DOVO (Belgian Defence Department for clearing up and destroying explosives) was engaged. The activities were interrupted several times because of needing to secure a third non-detonated bomb in hall 1 (Zone 4). The two forensic pathologists resumed their activities the next day (Day 1) between 8:30 am and 1:00 pm. The assistant in forensic medicine joined the PM teams at the morgue to proceed with the autopsies.

**Figure 1.**
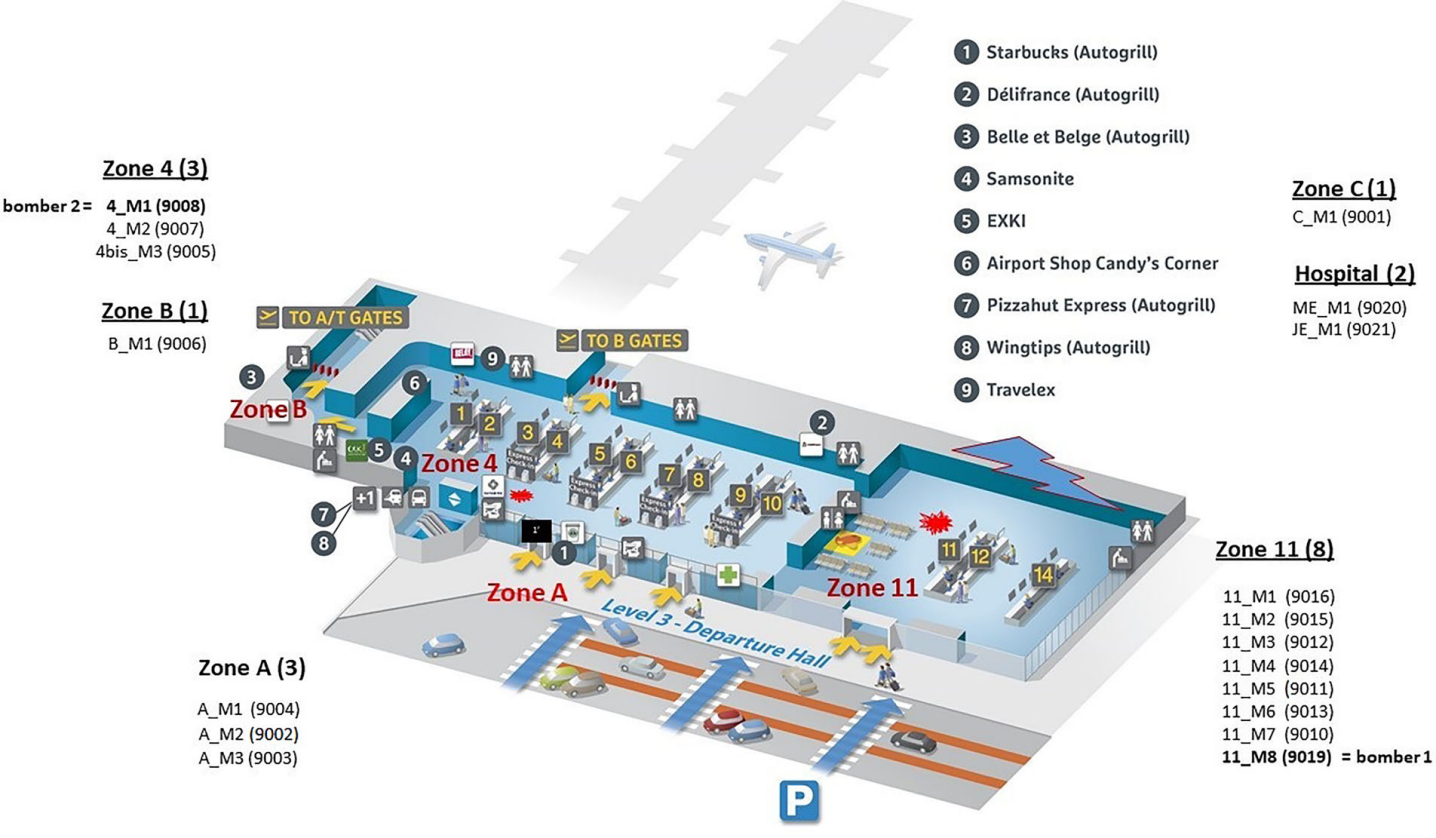
Overview of the scene (recovery of the corpses).

Investigators determined that there were two largely separated explosion areas in the departures hall (Figure 1). The first explosion (at 7:58 am) was in hall 2 near the Delta Airlines check-in counter with an explosion crater of 30 cm in diameter and 3 m away from a children’s playground (designated as “Zone 11”). The second explosion (at 7:59 am) was in hall 1 near the statue “Flight in Mind” and Starbucks coffee bar (designated “Zone 4”). The “kiss & ride” zone before the hall entrances was named “Zone A”, the corridor between Zone 4 and the A gates was named “Zone B”, and the temporary morgue at the fire station was named “Zone C”.

On Day 0, the bodies of 13 victims and one (alleged) bomber were recovered at the scene: three in Zone A (two at the entrance of hall 2 and one near the entrance of hall 1), three in Zone 4 including the second bomber, one in Zone B, and seven in Zone 11. Finally, the severely mutilated corpse of the supposed first bomber was discovered the next day (Day 1) in the debris of a collapsed wall in Zone 11. One corpse was transferred into the temporary morgue (Zone C).

**Figure 2.**
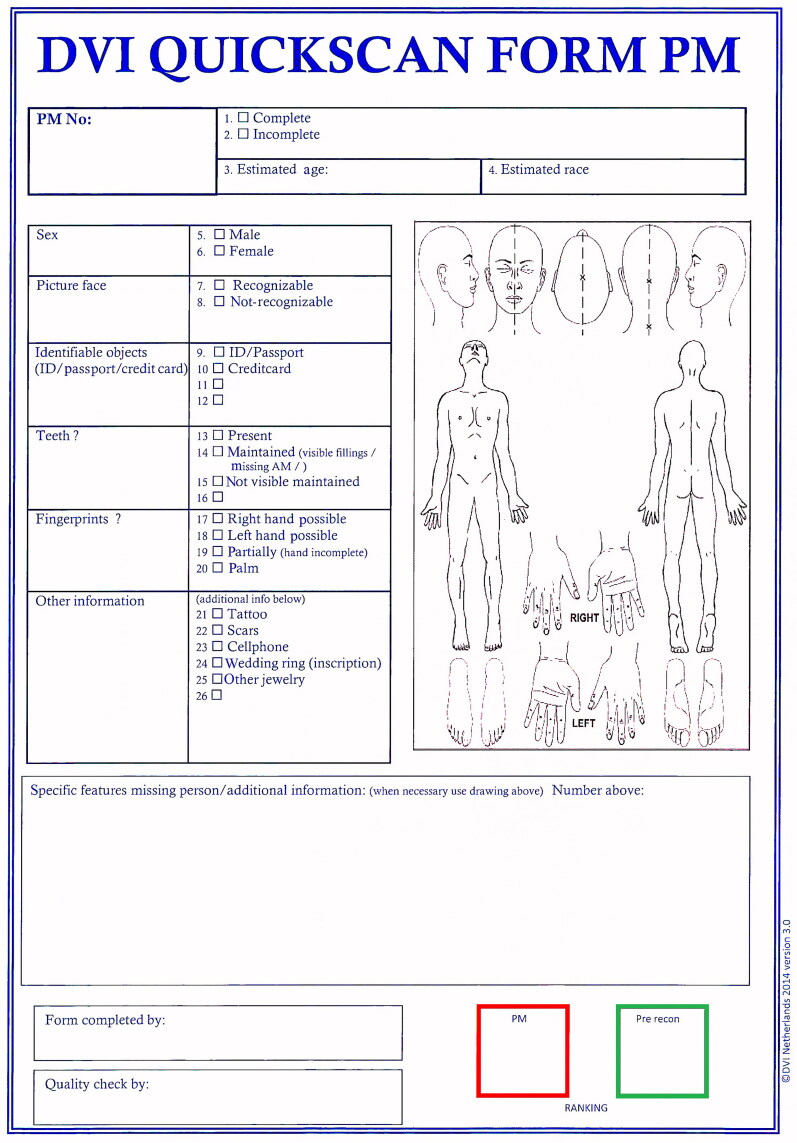
Quickscan postmortem form.

All recovered bodies (14 victims and two alleged bombers) first underwent a superficial external examination in the field and findings were noted using the quickscan form (Figure 2). Eight victims carried personal documents that included their names. A separated lower leg was also found at the scene, which was later revealed to have come from an on scene amputation of a survived victim.

The 15 bodies recovered on Day 0 were transferred to the hospital morgue throughout that afternoon and evening. The copse of a bomber was only recovered and transferred the next day. Two other still unidentified victims died in the hospital and were transferred to the morgue over the next 2 days.

#### PM investigations at the morgue

**Figure 3.**
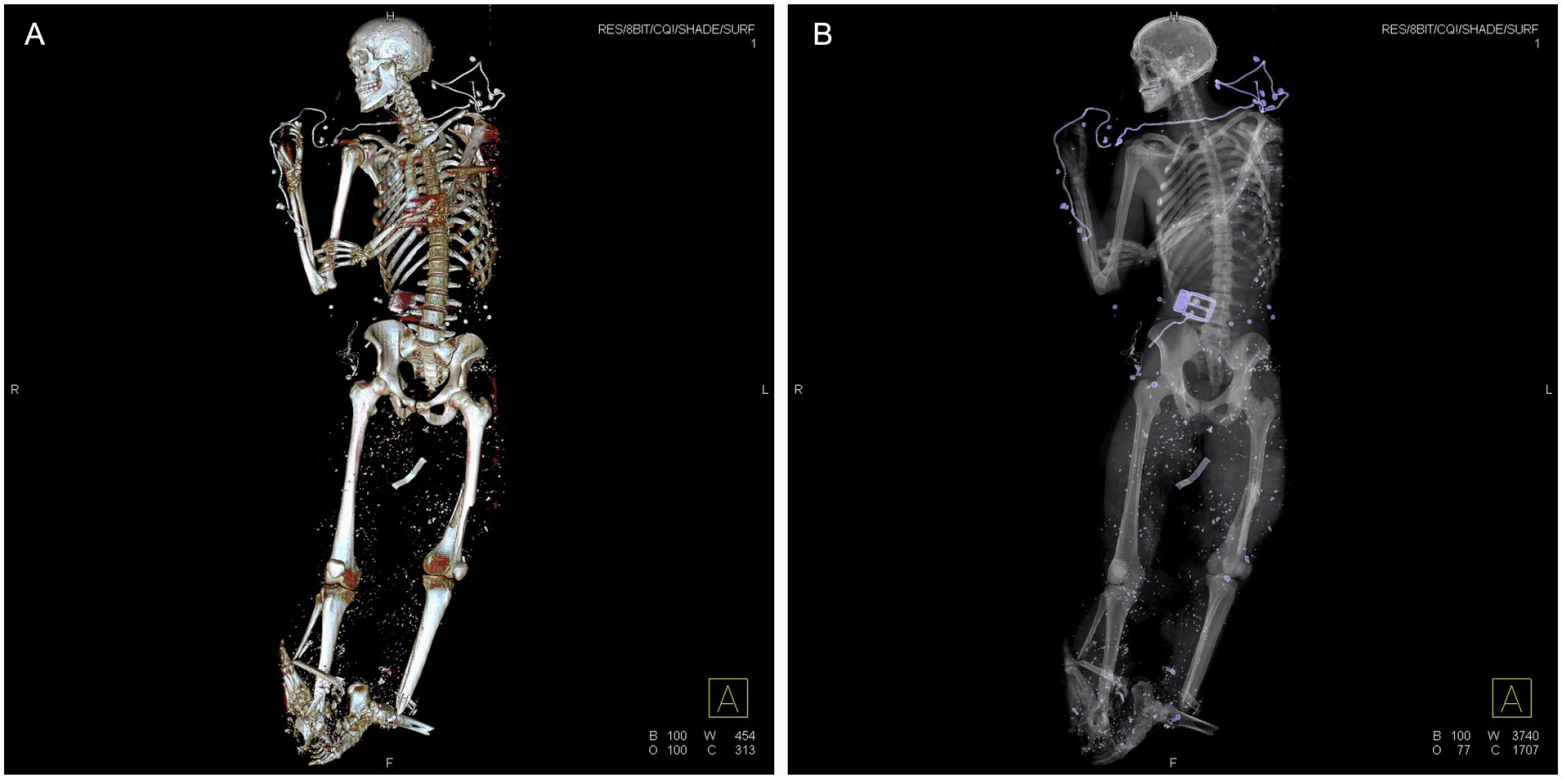
Postmortem CT-scan of victim: penetrated foreign objects (shrapnel) and partial avulsion underlegs. (A) Three-dimensional image of total skeleton. (B) Conventional radiologic overview of total body.

Meanwhile, the judge taking over the criminal investigation assigned our group to complete victim identification in collaboration with the DVI (final group responsible for the formal identification process in Belgium), perform autopsies on all bodies to determine the cause of death, and gather forensic evidence. The assignment was fulfilled following our standard procedures concerning forensic autopsy (ISO 17020), including sampling, histology, PMCT-scan (Figure 3), and identification. All morgue and department administration personnel were engaged in these processes. PM investigations were performed on a total of 16 victims and two suicide bombers.

Three bodies that arrived at the morgue on Day 0 in the afternoon underwent PMCT and a full forensic autopsy, including forensic odontological examinations. Another 12 bodies arrived during the evening and PMCTs were performed throughout the night. The last recovered corpse was examined the next Day 1. The two victims initially transported to hospitals were finally examined the following days.

On Day 1, between 8:30 am and 5:30 pm, one identification chain began in the first autopsy room. This included an external forensic pathologist and their trainee, a forensic odontologist, members of DVI to fill in the PM form, and scientific police to document the findings (including personal belongings and clothing). These external examinations, followed by an odontological examination in a separate room, were completed on 13 victims. For three of them that were externally examined in the morning, the examinations were completed with a full forensic autopsy in the second autopsy room in the afternoon (until 6:00 pm). The most experienced forensic pathologist (WVdV) and doctor assistant performed the same day PM examinations on both the body remnants and parts of the alleged suicide bombers. At the end of Day 1, all INTERPOL PM forms were completed for all but the internal findings and were handed over to the DVI.

On Day 2, forensic autopsies on all remaining (and previously externally examined) 10 victims were performed by two teams each composed of a forensic pathologist supervisor and an experienced trainee. At 4:30 pm, all PM investigations of the 16 victims and the two perpetrators were accomplished. The identities of the two bombers were suggested by digital fingerprint analysis on Day 1 and confirmed on Day 4 by DNA analysis.

Ten victims were formally identified in the late evening of Day 2 using the INTERPOL procedure for comparison of PM and AM forms in a reconciliation report signed by the two forensic pathologists engaged in the crime scene investigation and in the possession of the autopsy findings, the odontologist, and the DVI. On Day 3, the identity of another victim was established. The last two victims were formally identified on Day 6. The identifications in 14 cases relied on odontology and secondary characteristics, and in two cases on DNA (the last result obtained on Day 5). Body dress was completed and the dignified farewell procedure was started on Day 3. Eight different nationalities were represented among the 16 victims: Belgium (5), USA (4), the Netherlands (2), China (1), France (1), Germany (1), Peru (1), and Sweden (1).

#### PM findings

**Figure 4.**
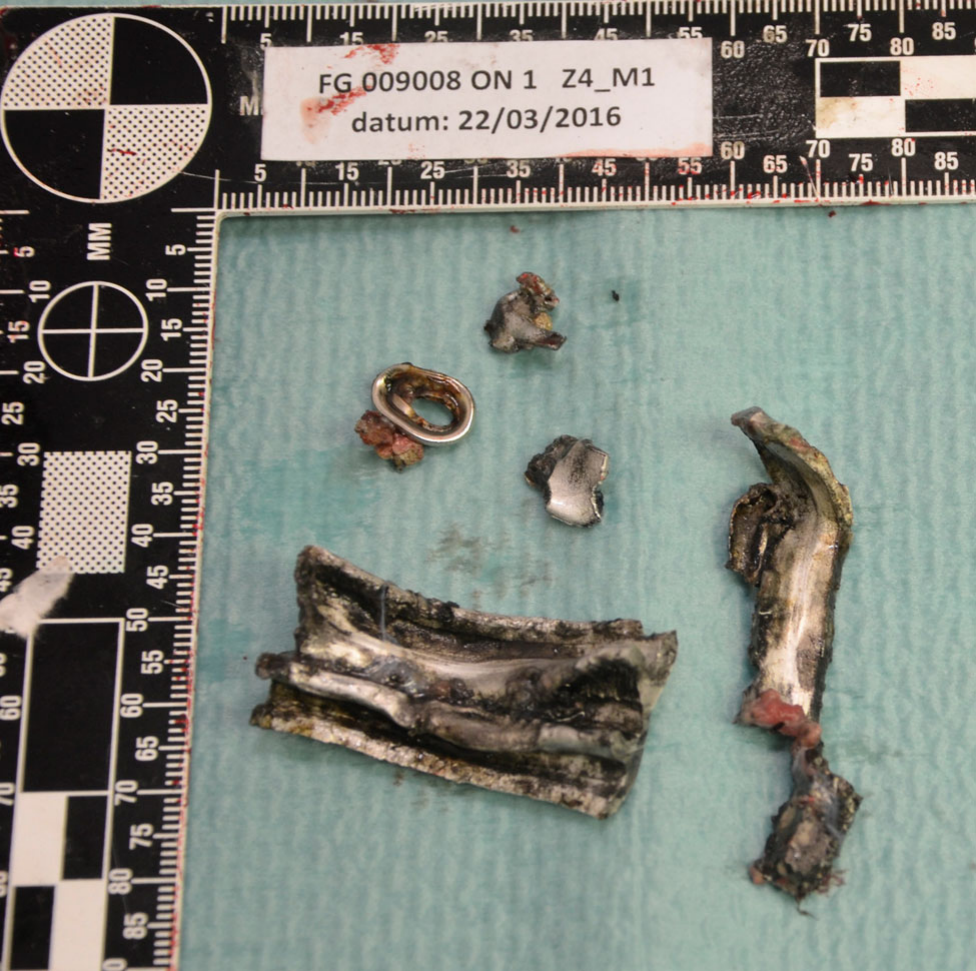
Removed shrapnel (undefined metal devices, probably scrap).

**Figure 5.**
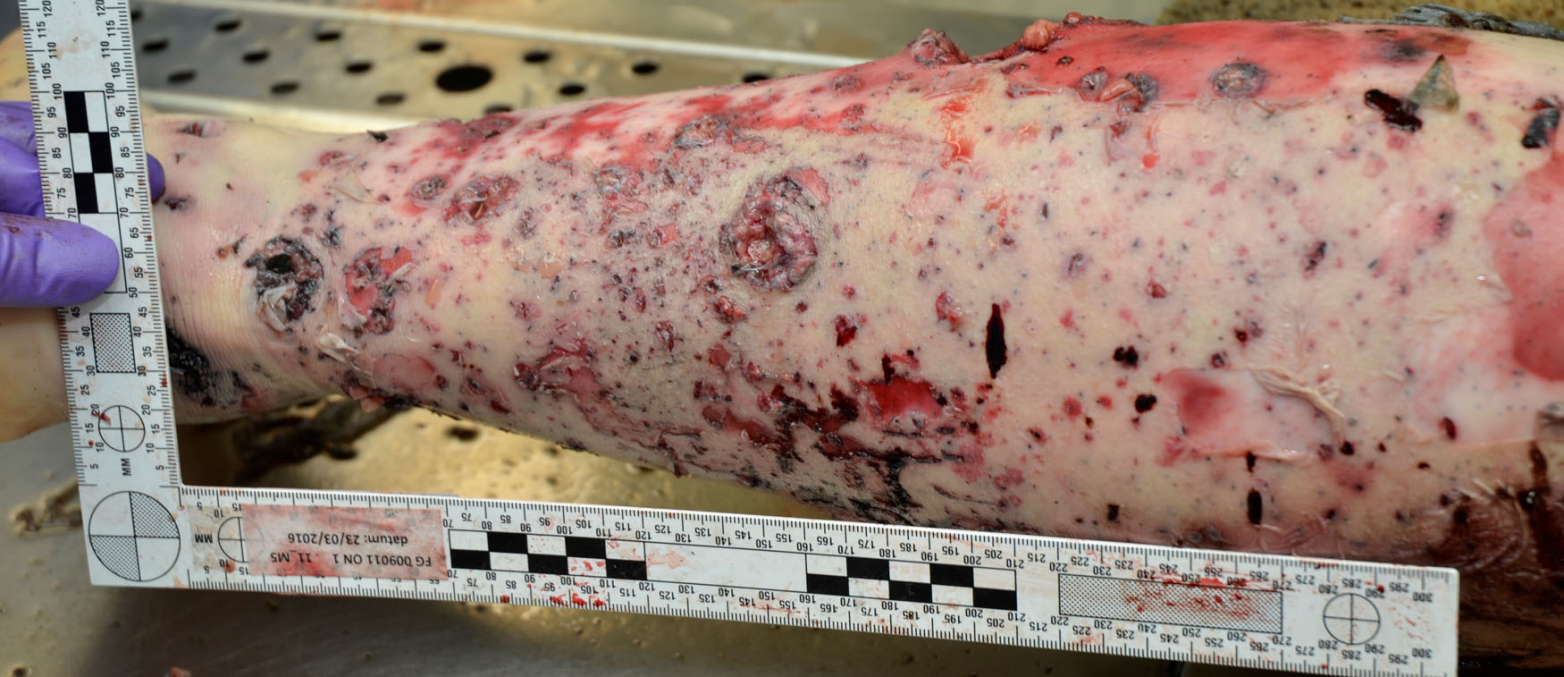
Secondary trauma: penetrating missile injuries and peppering.

Internal autopsy findings could contribute to the identification of six victims, such as the absence of a testicle, hip prosthesis, absence of uterus and ovary (post *hysterectomia totalis* et *radicalis*), congenital agenesis of the left kidney, ocular prosthesis, intravaginal contraception ring, and intrauterine contraceptive device. Twelve of the 14 victims who died on the scene had severe polytrauma and could be located within a few meters of the explosion centre. Using the distinction between primary, secondary, tertiary, quaternary, and quinary lesions [9], the lesions could in all cases be classified as primary blast and secondary penetrating missile wounds [10–12] following the discovery of large amounts of metal devices such as screws and metal plates of several centimeters long (corresponding to the exploded nail bombs). Many of these shrapnel fragments were seized as physical evidence (Figures 4 and 5).

All nine victims associated with the first and most forceful explosion in hall 2 (Zone 11) died from perforating craniocerebral trauma, mostly with complex skull fractures. Blast lung was seen in six, partial amputation of extremities (three lower legs, one thigh, one foot, three forearms, one thumb) in seven, and mostly complex open and displaced fractures of the legs in six, in addition to penetrating missile injuries (with peppering or polycribblage) as well as flash burns in all victims.

Thoracic and abdominal perforating trauma were seen in two and four cases, respectively. Severe musculocutaneous and skeletal injuries of the legs were a prominent feature, and are typically seen in war and terroristic bombings [13, 14]. This is in contrast to civil gas explosions, and there is currently no established explanation for this preponderance of leg lesions except in the case of landmines [15, 16]. The victim in Zone C (temporary morgue at the airport) had a tourniquet placed around the left thigh because of partial amputation of the lower leg. Furthermore, this injury pattern was consistent with those of the other victims found in Zone 11.

**Figure 6.**
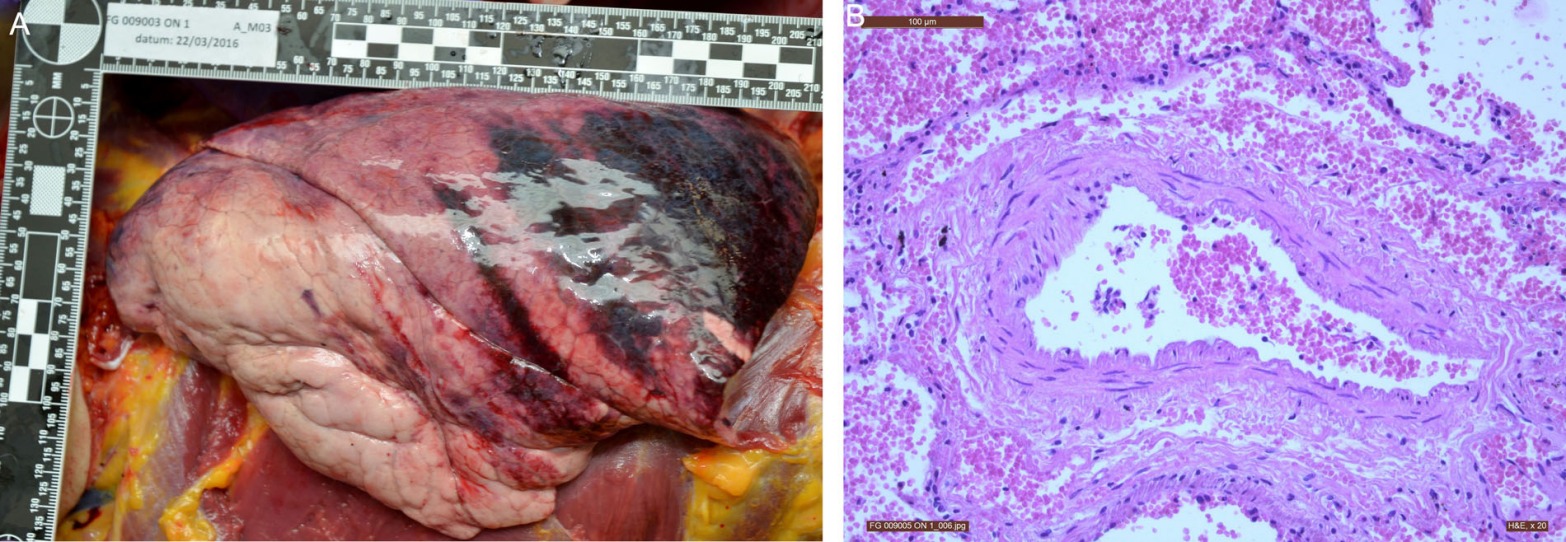
Blast lung injury visualized by (A) macroscopy, and (B) microscopy (HE, ×20): typical perivascular cuff-like haemorrhages.

The two victims recovered from the second bomb area (Zone 4) succumbed to destructive craniocerebral trauma in addition to flash burns and severe musculocutaneous and skeletal injuries in the legs. One of these victims also had thoracic and abdominal blast injuries (Figure 6). The missile injuries were less severe. The distribution and type of the previously mentioned injuries are consistent with the pattern typically observed in closed room and half-open space explosions [17].

The only victim in Zone B, between the second bomb area and the gates, was seen on surveillance video falling down at the moment of and at distance from the second explosion in Zone 4. She succumbed after crawling over several meters, leaving behind an intense blood smear. She died from a single deep perforating injury (8 cm × 6 cm laceration) of the left dorsomedial side of the pelvis by a 12 cm × 3 cm metal device, which severed the great iliac vessels. No other lesions were found.

An older individual with significant arteriosclerosis, cardiac dilatation, and left ventricular hypertrophy showed no major internal injuries except for fractures of the mandibula and one rib, in addition to minor missile injuries in the legs. The cause of death was considered to be an acute stress or blast-related cardiac dysrhythmia [18, 19]. Two victims died in the hospital: one from a subtotal amputation of the right leg at knee level with otherwise only a few minor missile injuries in the legs, and the other from a laceration of the right femoral artery caused by missile injuries restricted to the right leg.

The bodies of both bombers were partially disrupted. Bomber 1 (Zone 11) had complete craniocerebral destruction and amputation of all four extremities, which resulted in a multiple lacerated trunk and organs. Bomber 2 (Zone 4) was severely burned and partially carbonized with disruption of the abdomen, complete avulsion of the right pelvis and hip (recovered from the ceiling above the explosion centre), and complete skin avulsion of the right leg and left thigh. There was also complete amputation of the leg above the knee. DNA analysis linked 102 body fragments recovered from the scene to bomber 1 (Zone 11) and 81 to bomber 2 (Zone 4). The findings at the scene suggested that bomber 1 was bent over the bomb and bomber 2 was carrying the bomb on his lap or was inclined with his lower body near the device when it exploded. Interestingly, toxicological analysis of the blood, brain, and lung revealed the presence of acetone peroxide in both bombers (in contrast to the absence in control samples of a victim), suggesting application of the explosive triacetone trioxide (TATP). The number of victims and the PM findings are consistent with the first explosion in hall 2 (Zone 11) being more forceful than the second in hall 1 (Zone 4), and with most victims being in the direct vicinity (within meters) of the explosion centres.

#### Examination of the body fragments

On Day 2 and 3, two forensic pathologists assisted with the recovery of body parts and fragments which were numbered and transferred to the morgue. A total of 706 body parts and fragments were inventoried, analysed and described with the help of two forensic anthropologists. DNA analysis was performed on 581 samples from 332 body parts or fragments, selected by size (>5 cm). Thirty-two body fragments (besides muscular and cutaneous fragments, especially fingers and brain tissue) were linked to four victims of the first explosion (Zone 11), two victims of the second explosion (Zone 4), and to the victim in the temporary mortuary (Zone C); 183 parts or fragments belonged to the two bombers. Ultimately, all identification items were primordial. A shredded hand and facial skin were also found, which contributed to the identification of one of the bombers. The body fragments were also compared with the unidentified bodies autopsied in the forensic laboratory rooms.

## Results

The restitution of the bodies was carried out only after a formal identification was established during the process of data reconciliation. This was allowed by the data matching collected by the two teams (AM and PM) and after DNA analysis, if necessary. This procedure follows the rules edited by the TTVI (Thai Tsunami Victim Identification) [20, 21].

As mentioned, four identification’s levels have been described [6, 8, 22–25]. We use them as defined as the most appropriate identification’s classifications:

- Formal identity (radiological, odontological, fingerprints, DNA comparisons);
- Probable identity (identity papers, tattoos, compatible dental formula);
- Possible identity (scars, pathological history);
- Excluded identity (incompatible dental formula, antecedent of incompatible fracture).

## Discussion

The recent activities of the Belgian DVI teams in identifying victims of terrorist acts are quite similar to previous operations (e.g. in Kosovo, by the end of the armed conflict between the Atlantic Alliance and the Serbian forces). They are especially similar in regard to the difficulties of on-site management and the recovery of bodies, as well as the identification techniques and the release of bodies to the relatives. However, the state of the bodies in Kosovo was different from that in Zaventem and in Brussels because, during our previous missions in 1999 and 2000, the bodies were mostly skeletonized with putrefied organic tissues. The identification methods were therefore slightly different in that we noted the biological components, such as sex, age, size, and certain pathological characteristics, based on the skeletons.

We still insisted on the major role concerning the technical and human difficulties of the AM team (especially with contacting the relatives of the deceased person). The dangerous nature of the PM team intervention must also be highlighted. They were faced with explosives devices during both the attacks in Brussels and during the missions in Kosovo.

In our experience, autopsies are a critical criminal investigation tool in terroristic attacks by contributing to identification of individuals, determining their exact cause of death, and assisting with the gathering of physical evidence. This information is also significant in grief conversations with the next of kin. Recently, increased attention has been given to the families of the missing persons by increasing attention to them and providing more additional support in the mourning process. Specialized police officers work towards restitution of victims’ personal objects to their families. Other forensic teams and their mortuary technicians aim for restoration of the bodies to release them to their loved ones in the best manner possible.

In general, the authorities of the concerned country provide their own recommendations regarding body fragment management and analysis. DNA analysis is performed if the body part meets a minimal length. We believe that the length of the body part should not be the only criteria here because, for example, DNA profiles can be established with only a single nail fragment. Additionally, we know that a single fragment can be the only remaining residue of a missing person in cases of explosion with highly fragmentation, especially in air crashes and gas explosions. In addition to the length, we should also consider the preservation status of the fragment and the type of tissue (e.g. bone, vascular patchwork, muscle, nail, etc.).

In conclusion, DNA samples should be collected from fragments that are at least 5 cm in length, but additional criteria should be developed. These should include a new classification regarding the potential quality of the sample to promote more rational and efficient decisions about the management of these analyses.
